# Draft Genome Sequence of *Streptomyces* Strain SJ1-7, a Soil Bacterial Isolate

**DOI:** 10.1128/MRA.01283-20

**Published:** 2021-03-11

**Authors:** Won-Jae Chi, Da Som Kim, Soonok Kim, Eu Ddeum Choi, Sook-Young Park

**Affiliations:** a Genetic Resources Assessment Division, National Institute of Biological Resources, Incheon, South Korea; b Department of Plant Medicine, Sunchon National University, Suncheon, South Korea; University of Arizona

## Abstract

The draft genome sequence of *Streptomyces* strain SJ1-7, a bacterial strain isolated from the rhizosphere of a Pinus densiflora plant, is reported. The whole-genome assembly comprised 7.9 Mbp, with a GC content of 71.80% and 4,262 predicted protein-coding genes.

## Abstract (precis)

The draft genome sequence of *Streptomyces* sp. strain SJ1-7, a bacterial strain isolated from the rhizosphere of a Pinus densiflora plant, is reported. The whole-genome assembly comprised 7.9 Mbp, with a GC content of 71.80% and 4,262 predicted protein-coding genes.

## ANNOUNCEMENT

The genus *Streptomyces* belongs to the order *Actinomycetales*, phylum *Actinobacteria*, and consists of diverse aerobic, Gram-positive filamentous bacteria abundant in soil. It is well known that *Streptomyces* spp. produce numerous valuable biologically active secondary metabolites (SMs), including antifungals (1), antibacterials (2), antivirals (3), antitumor agents (4), immunosuppressants (5), and plant growth-promoting factors (6). *Streptomyces* spp. have been reported to produce almost 8,000 SMs (7, 8). Considering that we have not fully explored its diversity and biochemical capabilities, the genus will likely offer more pharmaceuticals as well as compounds that can help agricultural industries (9–11).

*Streptomyces* strain SJ1-7 was isolated by single-colony isolation on starch-casein agar (SCA) medium from rhizospheric soils surrounding the roots of a Pinus densiflora plant in Sangju, South Korea (36°26′41.0″N, 128°15′16.9″E), in 2018. For long-term preservation, the purified colonies were scraped from agar plates using a scraper with 15% glycerol buffer solution, transferred to a sterile cryovial, and then stored at −80°C. The strain was incubated in SCA medium for 7 days at 28°C, and a single colony was incubated in casein-starch-peptone-yeast extract-malt extract (CSPY-ME) broth with shaking at 120 rpm at 28°C for 7 days (12). Genomic DNA from SJ1-7 was extracted using a MagAttract high-molecular-weight DNA kit (Qiagen, Hilden, Germany) as instructed by the manufacturer. The extracted genomic DNA was cleaned using a phenol-chloroform-isoamyl alcohol (25:24:1) extraction protocol (13). Purified genomic DNA (15 μg) was size selected (15 to 20 kb) using a Covaris g-TUBE device and checked for concentration (Qubit). For genome sequencing, a single-molecule real-time (SMRT) cell library was prepared according to the Pacific Bioscience sample preparation protocol (https://www.pacb.com/wp-content/uploads/2015/09/User-Bulletin-Guidelines-for-Preparing-20-kb-SMRTbell-Templates.pdf), and sequencing was performed using the PacBio RS II platform by DNALink, Inc. (Seoul, South Korea). Sequencing generated 150,292 reads with a mean read length of 4,353 bp. The raw reads were trimmed, filtered, and assembled using the Hierarchical Genome Assembly Process (HGAP) v. 3.0 pipeline with default parameters as embedded in the PacBio SMRT Link software. The genome assembly yielded two contigs with 7,764,317 bp and 157,044 bp, representing 54.29-fold coverage and a 71.5% GC ratio. A total of 8,622 coding sequences (CDSs) and the genes for 18 rRNAs and 76 tRNAs were predicted by the Prokka v. 1.13.3 annotation pipeline with default parameters (14). Further analysis of the genome sequence, including functional and biochemical analyses, will reveal its secondary metabolism-associated genes. This draft genome sequence will also serve as a reference for comparative genomics with other *Streptomyces* spp.

### Data availability.

The whole-genome shotgun data set was deposited at GenBank under whole-genome sequencing project number JABFHJ000000000, BioProject accession number PRJNA623260, and BioSample accession number SAMN14543207. The version described in this article is the first version (JABFHJ010000000).
